# Targeting Aggrephagy for the Treatment of Alzheimer’s Disease

**DOI:** 10.3390/cells9020311

**Published:** 2020-01-28

**Authors:** Sandeep Malampati, Ju-Xian Song, Benjamin Chun-Kit Tong, Anusha Nalluri, Chuan-Bin Yang, Ziying Wang, Sravan Gopalkrishnashetty Sreenivasmurthy, Zhou Zhu, Jia Liu, Chengfu Su, Senthilkumar Krishnamoorthi, Ashok Iyaswamy, King-Ho Cheung, Jia-Hong Lu, Min Li

**Affiliations:** 1 Mr. and Mrs. Ko Chi Ming Centre for Parkinson’s Disease Research, School of Chinese Medicine, Hong Kong Baptist University, Hong Kong, China; deepu.pharma08@gmail.com (S.M.); juxian.song@gmail.com (J.-X.S.); benjamintck@gmail.com (B.C.-K.T.); anusha.nalluri1111@gmail.com (A.N.); nkyangchb@gmail.com (C.-B.Y.); Wangziying.12@163.com (Z.W.); sravangs@gmail.com (S.G.S.); zzhou1022@gmail.com (Z.Z.); liujiatheone@hotmail.com (J.L.); senthilnslab@gmail.com (S.K.); ashokenviro@gmail.com (A.I.); kingho@hkbu.edu.hk (K.-H.C.); 2 Medical College of Acupuncture-Moxibustion and Rehabilitation, Guangzhou University of Chinese Medicine, Guangzhou 510006, China; 3 State Key Laboratory of Quality Research in Chinese Medicine, Institute of Chinese Medical Sciences, University of Macau, Macao, China; jiahonglu@um.edu.mo

**Keywords:** aggrephagy, selective autophagy, Alzheimer’s disease, aggregates

## Abstract

Alzheimer’s disease (AD) is one of the most common neurodegenerative diseases in older individuals with specific neuropsychiatric symptoms. It is a proteinopathy, pathologically characterized by the presence of misfolded protein (Aβ and Tau) aggregates in the brain, causing progressive dementia. Increasing studies have provided evidence that the defect in protein-degrading systems, especially the autophagy-lysosome pathway (ALP), plays an important role in the pathogenesis of AD. Recent studies have demonstrated that AD-associated protein aggregates can be selectively recognized by some receptors and then be degraded by ALP, a process termed aggrephagy. In this study, we reviewed the role of aggrephagy in AD development and discussed the strategy of promoting aggrephagy using small molecules for the treatment of AD.

## 1. Introduction

Alzheimer’s disease (AD) is the most prevalent neurodegenerative disorder in the world. It is clinically characterized by memory dysfunction; it is pathologically characterized by beta-amyloid (Aβ) and tau toxic aggregates [1] in the cortex and hippocampus of the brain. Senile plaques composed of fibrillar Aβ and neurofibrillary tangles (NFTs) formed by hyperphosphorylated tau (p-Tau) proteins are currently considered as the main pathological markers of AD [2,3]. This AD pathology appears to be associated with the mutated gene inheritance (familial AD) in early-onset AD or caused by the uncertain genetic or environmental factors (sporadic AD) in the most prevalent late-onset AD cases [4]. In familial AD mutation, the normal non-amyloidogenic cleavage of amyloid precursor protein (APP) involving α-secretase and γ-secretase is shifted to the Aβ producing, abnormal amyloidogenic pathway involving β-secretase and γ-secretase, and the generated Aβ self-aggregates into oligomers and fibrils [5,6,7]. Autosomal dominant inheritance of APP gene mutation, as well as γ-secretase components Presenilin-1 and Presenilin-2 gene mutations are strongly correlated with Aβ pathology [8,9]. According to the amyloid cascade hypothesis, Aβ production and its oligomer formation is the main AD pathological event and it is followed by NFT formation [10]. NFTs are formed due to neuronal microtubule-stabilizing protein tau hyperphosphorylation [11]. The irregular phosphorylation of tau prevents it from stabilizing microtubules and thereby induces it to undergo self-aggregation [12,13]. The impaired degradation of these aggregation-prone proteins leads to cytotoxicity, neuronal atrophy, neurodegeneration, and, ultimately, synaptic impairment and memory deficits [14,15].

**Figure 1:**
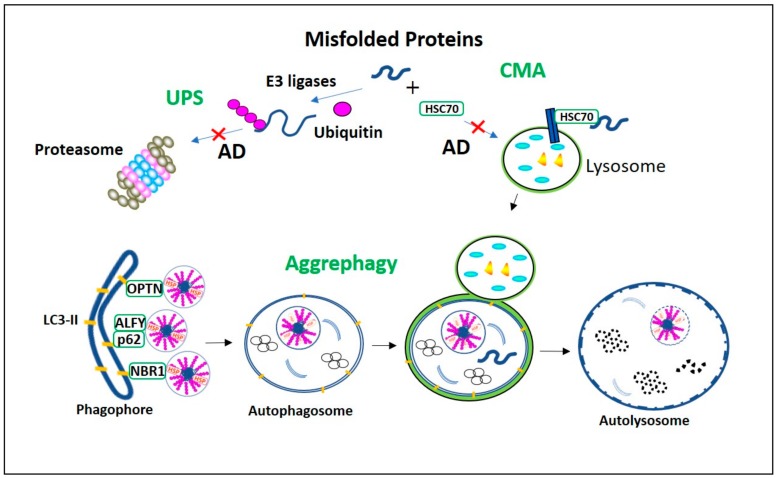
Misfolded proteins degradation processes in Alzheimer’s Disease (AD). Ubiquitin-proteasome system (UPS) and chaperone-mediated autophagy (CMA) proteolytic pathways are vulnerable to AD aggregates, and their impairment activates aggrephagy. The misfolded proteins with the KFERQ motif are recognized by HSC70 and recruited directly into lysosome via LAMP2a for degradation in CMA. In UPS the ubiquitinated misfolded proteins are degraded in proteasome. However, in AD, as these constitutive proteolytic pathways are vulnerable to the AD aggregates, aggrephagy is initiated. In aggrephagy, ubiquitinated aggregates, as well as their aggresomes are recognized by the aggrephagy receptors p62, Optineurin (OPTN), and neighbor of BRCA1 gene 1 (NBR1) and recruited eventually into the LC3-II containing double-membranous autophagosome. Further, this autophagosome fuses with the lysosome to form autolysosome and degrades these AD aggregates.

How do cells, especially neurons, rid themselves of the aggregation-prone proteins? The ubiquitin-proteasome system (UPS), chaperone-mediated autophagy (CMA), and the autophagy-lysosomal pathway (ALP) are the main cellular processes responsible for this function [16]. UPS and CMA degrade short-lived soluble proteins. Large, long-lived insoluble protein aggregates can only be degraded by macroautophagy (hereafter referred to as autophagy). Autophagy was initially considered to be a bulk degradation system [17]. However, increasing evidence indicates that autophagy can be highly specific, mediated by some receptors which recognize the substrates for degradation [18]. The term aggrephagy was introduced to describe the selective clearance of protein aggregates by autophagy [19]. In this review, we will discuss recent advances in understanding aggrephagy impairment in AD and in purposefully targeting aggrephagy with small molecules as a strategy for the prevention and treatment of AD (Figure 1).

## 2. Process and Regulation of Aggrephagy

### 2.1. Aggresome Formation

The selective autophagic clearance of aggregated proteins is called aggrephagy [17]. In proteinopathy, hydrophobic interaction in-between the aggregation-prone proteins lead to the formation of aggregates. In general, the amino acid sequence of a protein determines its post-translational modifications [20]. Post-translational modifications, such as protein folding, not only regulate the protein function but also mask the hydrophobic regions in the newly synthesized protein [20,21]. However, events such as intense protein overexpression, gene missense mutation, incomplete protein synthesis, endoplasmic reticulum stress, and protein folding co-factors shortages may cause unmasking of these hydrophobic protein regions [22]. Intra-protein interaction between the exposed hydrophobic areas results in misfolded proteins. Interaction of exposed hydrophobic regions between multiple misfolded proteins leads to the creation of protein aggregates. Once a protein aggregate is formed, its exposed hydrophobic regions will further recruit misfolded proteins until its hydrophobic regions are covered [23,24]. These aggregates cause impaired neuronal membrane permeability, irregular calcium homeostasis, inflammation, oxidative stress-induced neurotoxicity, and physiological abnormalities [15,25,26]. Healthy neurons can counteract this process by packing misfolded proteins into early aggresomes, which are membrane-free, large insoluble structure located near the nucleus [27]. These early aggresomes are cytotoxic and they are surrounded by intermediate cytoskeleton filaments. In the later stage, aggresomes are processed into non-toxic double-membrane autophagosome and degraded in the lysosome [28,29,30,31]. 

### 2.2. Aggrephagy Regulation

The pathophysiology of proteinopathy is directly related to detergent-soluble misfolded proteins long term aggregation into devastating detergent-insoluble aggregates [32]. Heat shock proteins (HSPs), constitutive chaperone-mediated autophagy (CMA), and the ubiquitin proteasomal system (UPS) halt the detergent-soluble aggregation-prone protein level elevation [16]. HSP60, HSP70, and HSP90 can bind to misfolded proteins to refold them. They can also neutralize the exposed hydrophobic regions in the misfolded proteins and stop their aggregation process [33]. In the CMA pathway, heat shock cognate protein 70 (HSC70) binds to the KFERQ motif of the misfolded protein and delivers it directly to a lysosome via LAMP2a for degradation (Figure 1) [34]. In UPS and canonical autophagy, the ubiquitination of the aggregates is vital for their degradation [35]. Ubiquitin contains seven lysine residues (Lys6, Lys11, Lys27, Lys29, Lys33, Lys48, and Lys63) [36]. These lysine residues covalently bind to the aggregates and form polyubiquitinated chains that can be recognized for degradation [37]. In particular, Lys48-linked polyubiquitinated aggregates are specific for UPS degradation. The aggregates will be delivered to 26S proteasome and undergo ATP-dependent unfolding, followed by proteasome digestion (Figure 1) [38,39]. However, both UPS and CMA pathways are unable to deal with dense aggregates. Besides, UPS and CMA machinery are susceptible to aggregate processing, and an overwhelming protein load will saturate their aggregate-handling ability. In such a case, aggrephagy will be activated and serve as an alternative degradation mechanism [17].

### 2.3. Role of Aggrephagy Receptors

Sequestosome-1/p62, a neighbor of BRCA1 gene 1 (NBR1), and optineurin are aggrephagy-specific cargo receptors. These aggrephagy receptors act as binding bridges between polyubiquitinated substrates as well as autophagosomal microtubule light chain complex-II (LC3-II) and GABARAP [17]. Ubiquitin-binding domain (UBD) and LC3-interacting motif in both p62 and NBR1 are essential for aggrephagy receptor function. In both p62 and NBR1, UBD located in the C-terminal region specifically recognizes Lys63-linked polyubiquitin substrates and forms complex [40]. Simultaneously, the LC3-interacting motif in p62 and NBR1 promotes the delivery of complexes formed from p62 or NBR1 and polyubiquitinated aggregates to autophagosomes (Figure 1). Among these receptors, p62 is the only essential one for the regulation of substrate ubiquitination, and this ubiquitinated substrates lysosomal digestion. For example, it is suggested that p62 can influence TRAF6 (E3 ubiquitin ligase)-mediated polyubiquitination [41]. Also, p62 recruits a 400 KD autophagy linked FYVE (ALFY) nuclear protein into the cytoplasm for the autophagic degradation of aggregates. ALFY is crucial in facilitating interaction between p62-linked aggregates and autophagosome membrane-bound LC3 [42]. The ALFY C-terminal region has BEACH, FYVE, and WD40 domains, which are crucial to this peptide’s functional role in aggrephagy [43]. In particular, the binding of its WD40 domain to Atg5 is essential for ATG5-ATG12-ATG16L1 E3 ligase complex formation. Binding of its FYVE domain to PtdIns3P enhances phagophore formation, while its BEACH domain binds to the p62-aggregate complex and acts as a scaffold between LC3 in phagophores [44]. Under normal autophagy conditions, p62 and NBR1 aggrephagy receptors facilitate aggregate degradation (Figure 1). It has been demonstrated that under limited autophagy conditions, the impaired clearance of p62 and NBR1 promotes the formation of toxic oligomers and aggregates. These p62 and NBR1 co-localized toxic aggregates incorporate into the toxic aggregates and expand them [45]. Also, when proteasomal aggregate degradation is impaired, tripartite motif-containing 50 (TRIM50) can enhance aggrephagy. TRIM50 is an E3 ubiquitin ligase, and it is reported to increase the aggregation of polyubiquitinated substrates into aggresomes. It enhances aggrephagy by increasing p62 expression and also by influencing HDAC6-mediated misfolded protein retrograde axonal transport when proteasomal aggregates degradation is impaired [46]. 

### 2.4. Role of HDAC6 in Aggrephagy

**Figure 2:**
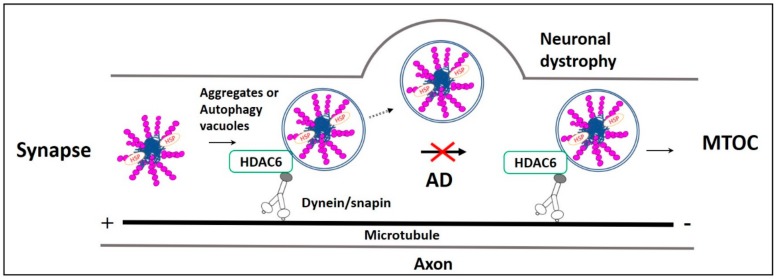
Fast axonal transport impairment in AD. Impairment in the microtubule-dependent fast axonal transportation of misfolded protein aggregates and autophagy vacuoles from the neuronal distal end to the lysosome rich microtubule-organizing center (MTOC) leads to neuronal dystrophy in AD. Usually, HDAC6 recognizes ubiquitinated AD misfolded proteins p-Tau, Aβ, their aggregates, and autophagy vacuoles. Then they are retrogradely transported by the axonal microtubule motor/adaptor complex dynein/snapin for autophagic degradation in MTOC. However, in AD, retrograde axonal transportation impairment increases neuronal autophagy stress, causing p-Tau and Aβ aggregates, as well as these aggregates, filled autophagy vacuoles fusion with the plasma membrane resulting in neuronal dystrophy.

Misfolded proteins generated in axons and dendrites are retrogradely transported to the lysosome-rich microtubule-organizing center (MTOC). In MTOC, they are packed into aggresomes, and subsequently degraded in the lysosome [47,48]. These functions are regulated by histone deacetylase-6 (HDAC6). HDAC6 is a deacetylating enzyme that is crucial in the microtubule- transport machinery [49]. In aggrephagy, HDAC6 deacetylates α-tubulin, cortactin, and HSP-90 [50]. Also, HDAC6 is actively involved in the sorting of polyubiquitinated misfolded proteins for axonal retrograde transportation that uses Dynein-snapin, a motor-adaptor complex [51]. Many other factors are involved in regulating HDAC6-associated aggrephagy. Protein kinase-2 (CK2) phosphorylates HDAC6 directly and regulates its deacetylation activity [52]. Aggregates polyubiquitinated by TRIM50, TRAF6, and parkin E3 ubiquitin ligases [53,54] are simultaneously deubiquitinated by ataxin-3 to generate diglycine motifs at ubiquitin C-terminuses to form recognition sites for HDAC6. HDAC6 recognizes the diglycine motifs of aggregates through its ubiquitin zinc finger domain (BUZ). Then HDAC6 recruits these aggregates to dynein motor complex through its dynein motor binding domain (DMB). The recruited aggregates are transported via a dynein-snapin axonal microtubule transport system and packed into aggresomes near MTOC (Figure 2) [55]. HDAC6-mediated LC3-II deacetylation is associated with starvation-induced autophagy induction and aggregates degradation [56]. It is found that p62 colocalizes and inhibits HDAC6 deacetylation. As a result, cortactin acetylation is promoted, which is necessary for cytoskeletal F-Actin network remodeling. F-Actin network remodeling enables autophagosome maturation and facilitates aggregate clearance [54].

## 3. Aggrephagy Dysfunction in AD

### 3.1. Aggresomes in AD

AD pathological proteins Aβ and p-Tau can form aggresomes. Lu et al. showed that cells overexpressing APP Arctic mutation (APP693G) resulted in APP amyloidogenic processing and increased Aβ-42 levels. Using super-resolution microscopy, they found that elevated Aβ-42 monomers progressively assembled into degradation-resistant aggresomes in the perinuclear space [57]. In general, ubiquitin binds to Aβ for proteasomal degradation [58]. Also, these polyubiquitinated chains on Aβ cause thermodynamic instability. As a result, they are not appropriately folded, making this Aβ resistant to proteasomal degradation. Impairment in proper Aβ folding alters their physicochemical properties and enhances Aβ fibrils and plaques formation. Altogether, Aβ is unfolded due to the polyubiquitination, and this unfolded Aβ aggregates into insoluble Aβ fibrils. In aggrephagy, polyubiquitinated Aβ peptides are subjected to autophagic degradation. These polyubiquitinated Aβ fibrils and aggregates selectively bind to UBA domains of p62 and NBR1 to initiate their degradation through aggrephagy [59]. On the other hand, the role of HDAC6 in Aβ aggresome formation is still unclear. Ran-binding protein-9 (RanBP9) levels have been found to be elevated in the AD patient brain [60]. It is proven that RanBP9 increases Aβ secretion by increasing the β-secretase activity [61], and it can also influence aggresome formation by interacting with HDAC6 [62]. However, there is no direct evidence elucidating HDAC6 and RanBP9 inter-relationship in Aβ aggresome formation and its autophagic degradation.

Clinically AD is expressed in the form of extracellular Aβ surrounded by the dystrophic neurites with intracellular NFTs [63]. NFTs are formed due to the aggregation of abnormally hyperphosphorylated tau into paired helical filaments (PHFs) [64]. p-Tau generated at the synapse are retrogradely transported to form aggresomes near the nucleus for lysosomal degradation [65]. It is reported that cells treated with human PHFs can internalize this extracellular tau and form vimnetin- and dynein-colocalized aggresomes near perinuclear spaces [27]. When proteasomal aggregates degradation is inhibited, the carboxyl terminus of heat-shock cognate (Hsc)70-interacting protein (CHIP) in association with HSP 70 regulates tau ubiquitination [66]. The ubiquitinated p-Tau is recruited by aggrephagy receptor p62 [65,67]. HDAC6 binds to p62 and promotes the p-Tau and p62 complex retrograde transport to form tau aggresomes [68]. Also, studies reported that HDAC6 responds to the proteostasis stress caused by proteasomal inhibition and re-organizes the cytoskeletal actin network to induce tau aggresome formation [69,70]. Studies reported that ubiquitin carboxy-terminal hydroxylase-L1 (UCH-L1) enzyme also influences tau aggresome formation. UCHL1 is a deubiquitinating enzyme, and it is abundantly expressed in the brain [71]. UCH-L1 inhibition results in tau hyperphosphorylation and also tau microtubule-stabilizing functional loss [72]. Moreover, UCHL1 can modulate HDAC6 activity and affects tau aggresome formation. UCHL1 inhibition reduces the interaction between p-Tau as well as HDAC6 and suppresses tau aggresome formation [73]. 

### 3.2. Dysregulation in Aggregates Retrograde Transport in AD

Quick autophagic degradation of AD pathological proteins is necessary for the maintenance of synaptic plasticity [74]. Failure in this dynamic process is one of the main reasons for altered synaptic plasticity and cognitive decline in AD [75]. Alterations in fast axonal transport are observed in different AD Drosophila [76] and mice [77,78] models. Aβ and p-Tau accumulation at synapses impairs synaptic neurotransmission and axonal retrograde transport [79]. Normally, p-Tau peptides produced at the synaptic region are recruited directly or as aggresomes into autophagosomes [65,80], and they are retrogradely transported to neuronal perinuclear space for aggrephagy- mediated lysosomal degradation [68]. Also, p-Tau aggresome formation requires microtubule motor/adaptor proteins dynein/snapin complex-mediated axonal retrograde transport [68]. But in AD, Aβ as well as tau affect both kinesin and dynein functions, which are necessary for axonal anterograde and retrograde transportation mechanisms, respectively [81,82,83,84,85]. Also, a study reported that Aβ oligomers present in the autophagic vacuole competitively inhibit dynein and snapin coupling and their complex formation. This complex is necessary for the fast axonal transport of aggregates and autophagy vacuoles to the perinuclear space for lysosomal degradation [86]. Impairment in their axonal transportation affects their lysosomal degradation [47]. Moreover, this impairment increases the aggregates level in neuronal axons and synapses. This increase reduces synaptic plasticity and promotes neurodegeneration [79]. Similarly, dynactin-P50, a dynein subunit expression increases with age, whereas dynactin-P50 expression is reduced and colocalized with Aβ plaques in AD patients carrying APOEε3,3 or APOEε4,4 mutation [87]. Also, microtubule anterograde transport protein Kinesin-1 knockout caused axonal transport impairment in APP overexpressed Tg_SWE_APP^prp^ mouse. These mice are observed with elevated Aβ42/Aβ40 ratio, causing autophagy vacuoles accumulation and axonal swelling [88]. Furthermore, these stranded autophagy vacuoles accumulated in axons are filled with AD pathologic proteins, and they fuse with the neuron plasma membrane [89]. This fusion results in membrane expansion, neuronal dystrophy, and neurodegeneration. Autophagy vacuole accumulation in distal end of neurons imposes neuronal autophagy stress in AD [47]. These synaptic autophagy vacuoles and aggregates clearance failure imposes synaptic toxicity resulting in impaired synaptic plasticity and memory dysfunction (Figure 2) [90,91]. 

CK2, protein phosphatase-1 (PP-1), and glycogen synthase kinase-3β (GSK-3β) kinases are actively involved in the axonal transport mechanism, and their functions in this transport mechanism are impaired in AD [92,93,94]. Janosine kinase-3 interacting protein-3 (JIP3) knockout in 5xFAD mice aggravates Aβ plaque pathology due to lysosomal accumulation in axons mimicking neuronal dystrophy. In these mice, accumulated lysosomes have increased levels of APP amyloidogenic processing enzymes, BACE1, and PS2 [95]. In another study, neurites associated with Aβ plaques were found to disrupt microtubule structure and elevate the BACE1 level [8]. Elevated BACE1 cleaves the APP present in the accumulated autophagic vacuoles and further raises the Aβ level [96]. Also, loss of nuclear receptor-binding factor-2 (NRBF2), an autophagy gene, results in APP-CTF accumulation and Aβ production [97]. Increased Aβ load in autophagic vacuoles further exacerbates autophagy stress and AD pathology. Aggravated AD pathology ultimately affects communication between neurons, as well as their normal function, and ultimately resulting in neuronal death [8,98].

### 3.3. Aggrephagy Receptor Impairment

Nuclear factor (erythroid-derived 2)-like factor (Nrf2) is a leucine zipper family protein. It regulates the expression of aggrephagy receptors p62 and NBR1 [99,100,101,102,103]. Also, it acts against aggregate-induced oxidative stress by increasing the antioxidant response element (ARE) expression. However, Nrf2 expression is found reduced in aged APP/PS1 mouse hippocampus, and due to this reduction, p62 and NBR1 expressions are also reduced [104]. Tripartite motif containing 16 (TRIM16), an E3 ubiquitin ligase regulates Nrf2 ubiquitination and enhances its stability by increasing the autophagic degradation of its negative regulator KEAP1. TRIM16 controls aggrephagy by enhancing the p62-KEAP1-Nrf2 signaling pathway [105]. In this pathway, KEAP1 interacts with Nrf2, and it acts as an adaptor protein for Nrf2 ubiquitination via cullin-3 ubiquitin ligase. Ubiquitinated NRF2 is degraded in proteasomes. But, p62 competitively binds to KEAP1 at the Nrf2 binding region and prevents its interaction with Nrf2. Hence, p62 binding to KEAP1 prevents Nrf2 ubiquitination and its proteasomal degradation [106]. TRIM16 inactivates KEAP1 by increasing its interaction with p62, and p62 facilitates KEAP1 autophagic degradation [105,107]. However, in an AD human brain temporal cortex, the KEAP1-interacting region of p62 is phosphorylated at ser-349 and ser-403 [108]. This abnormal phosphorylation of p62 impairs p62-KEAP1 interaction and results in decreased KEAP1 autophagic degradation. As a result, the KEAP1 level is increased. Activated KEAP1 in the cytoplasm enhances Nrf2 ubiquitination and degradation. The degradation of Nrf2 causes decreased expression of aggrephagy receptors p62, NBR1 and ARE. Reduction in aggrephagy receptors and AREs leads to less aggrephagy and more oxidative stress [105]. Similarly, another aggrephagy receptor NBR1 function may also be impaired due to elevated GSK3 levels in AD [109,110]. It is reported that GSK3 phosphorylates NBR1 at Thr-586 and prevents it from functioning as a polyubiquitinated cargo receptor in muscle cells [109,110]. However, the effect of elevated GSK3 on NBR1 function in AD has not been studied. Taken together, impairment in the expression and function of aggrephagy receptors leads to failure in aggregate degradation and increase of neurotoxicity in AD.

## 4. Aggrephagy as a Therapeutic Target for the Treatment of AD

### 4.1. Genetic Approach

**Figure 3:**
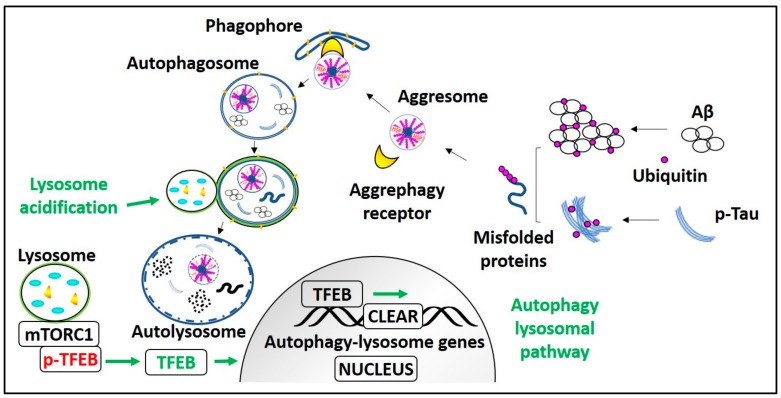
Process and regulation of aggrephagy in AD. mTORC1 holds Transcription factor EB (TFEB) on the lysosome membrane in phosphorylated form (p-TFEB). Aggrephagy is induced by mTORC1 inhibition or mTORC1 dependent/independent pathways that lead to TFEB activation, i.e., TFEB dephosphorylation. Dephosphorylated TFEB translocates into the nucleus, enhances CLEAR genes transcription, and promotes autophagy and lysosomal biogenesis. In AD, ubiquitinated monomeric Aβ and p-Tau peptides form misfolded proteins. These polyubiquitinated misfolded proteins are recognized by aggrephagy receptors and recruited into the LC3-II containing phagophore or autophagosome, which transforms into autolysosome after fusion with the lysosome and degrades AD pathological aggregates. Lysosome acidification failure due to PS1) mutation or elevated CTFβ levels can be treated with lysosomes targeted acidic nanoparticles.

Genetic manipulation of pivotal aggrephagy components and transcriptional activation of the autophagy lysosomal pathway (ALP) can improve the clearance of AD pathological aggregates. It is known that aggrephagy is impaired in AD, and HDAC6 promotes the aggrephagy of AD pathological substrates by transporting them to the lysosome rich perinuclear space. However, the HDAC6 expression and its acetylation activity are elevated in the AD patient’s cortex, and the hippocampus [111]. Moreover, in vitro studies demonstrated that HDAC6 downregulation reduces tau cluster formation, Aβ oligomer-induced neuroinflammatory stress and calcium dysregulation [112]. These results are also substantiated by another in vivo study showing that cognitive impairment is restored in HDAC6 knocked down APP/PS1 mice [113]. Hence, inhibition of HDAC6 acetylation activity is an effective therapeutic strategy for neurodegenerative diseases like AD. However, the HDAC6 acetylation inhibitors are anticipated not to affect the ubiquitinated substrates recognition ability of HDAC6 [114]. It is observed that the level of sarkosyl-insoluble tau is inversely related to the expression level of p-Tau ubiquitinating enzyme CHIP in JNPL3, a tau transgenic mice model. In JNPL3 mice, the sarkosyl-insoluble tau level in the cerebellum is less in comparison with the spinal cord region due to more CHIP expression in the cerebellum than the spinal cord. Moreover, in CHIP knockout mice, detergent-insoluble tau levels were elevated without altering the total tau level [115]. These results indicate that enhancing CHIP expression may promote pathological tau aggrephagy. Overexpression of the aggrephagy receptor p62 in APP/PS1 mice can improve cognitive dysfunction and reduce Aβ plaques level by enhancing Aβ autophagic degradation [116]. Also, the p62 chaperone activity promotes p-Tau solubility thus preventing detergent-insoluble NFT formation, because in the p62 knockout mice brains, Lys63-linked tau aggregates appear, and they are causally related to neurodegeneration and memory loss in these mice [117]. As Nrf2 regulates p62 and NBR1 expression, Nrf2 deficiency has been demonstrated to aggravate AD pathological symptoms [100,118], whereas its overexpression has alleviated both Aβ and p-Tau pathology [119,120]. Transcription factor EB (TFEB) is a master regulator of lysosomal biogenesis, and it also promotes aggrephagy. Much evidence indicates that TFEB overexpression causes profound enhancement of autophagy and induces autophagic clearance of AD aggregates [121,122,123,124]. TFEB is a self-transcription regulatory protein [125,126], and it also induces the expression of its gene promoter-peroxisome proliferator-activated receptor gamma co-activator1-α (PPARGC1α) [127]. Agents inducing PPARGC1α expression can reduce the AD aggregate level by activating TFEB and can also restore mitochondrial dysfunction [128,129]. Currently, we are working on small molecules that enhance autophagy lysosomal biogenesis by inducing the TFEB nuclear translocation. Our results indicate that TFEB activators have an excellent effect on reducing AD pathological aggregates as well as restoring cognitive impairment in mice models of AD (Figure 3) [130,131].

### 4.2. Lysosomes Functional Restoration with Nanotechnology

Lysosomal digestion of autophagy substrates is necessary for the survival of mature neurons [132]. Lysosomal hydrolase (e.g., cathepsin) activity and proteolysis indeed require optimal acidic pH [133]. In AD, presenilin-1 mutations and elevated CTFβ peptide levels impair lysosomal acidification and cathepsin activity, causing a massive pileup of autophagic vesicles with undigested autophagy substrates in neurons [134,135]. Presenilin-1 functional loss due to its mutation impairs the V-ATPase function, which leads to altered lysosomal Ca^2+^ homeostasis and results in defective lysosomal acidification and cathepsin D activity [136]. Also, amyloidogenic APP cleavage product CTFβ shifts lysosomal pH towards alkalinity and reduce cathepsin D activity [135]. These lysosome-related AD pathogenic events can be prevented with acidic nanoparticles. Studies show that these acidic nanoparticles accumulated in lysosomes and show prolonged lysosome functional restoration [137,138]. Specifically, in AD experimental models, treatment with poly-(dl-lactide-co-glycolide)-based acidic nanoparticles can reacidify lysosomes and restore cathepsin activity and autophagy function [135,136]. 

### 4.3. Small Molecule Aggrephagy Enhancers on AD Models

Small molecules that can (1) penetrate the brain, (2) induce aggregate clearance, (3) restore the obstructed axonal transport, as well as autophagy mechanism (4), ameliorate synaptic plasticity and (5) enhance cognitive function are invaluable for clinical AD therapy. Few small molecules have been reported to induce aggrephagy and reduce AD pathological aggregates by inhibiting the mammalian target of rapamycin (mTOR), as well as by the activation of TFEB (Table 1). 

#### 4.3.1. mTOR-Inhibiting Aggrephagy Inducers

mTOR regulates protein synthesis, cell proliferation, and growth. Depending on the availability of nutrition, it regulates the phosphorylation of its downstream targets P70S6K and 4EBP1 protein [139]. Growth factors and amino acids induce PtdIns3 kinase activity, phosphorylate AKT, and activate mTOR. The deficiency of these substances causes AMPK to phosphorylate the regulatory-associated protein of mTOR (RAPTOR) and generate binding sites for the 14-3-3 complexes that inhibit mTOR activity [140]. Under cell growth-promoting conditions, mTOR phosphorylates unc-51-like kinase 1 (ULK1), whereas, under conditions not favoring cell growth, ULK1 will be dephosphorylated and initiate autophagy [141]. The mTOR activity can be inhibited directly with rapamycin and torin1, or indirectly with wortmannin [142] and amino acid starvation [56,141,143,144,145]. Among these, rapamycin is a natural macrolide derivative reported to reduce AD pathological aggregates. Rapamycin treatment in AD mice has alleviated Aβ burden, improved memory dysfunction, and rescued cerebral amyloid angiopathy [144,146]. Rapamycin, as well as its prodrug form Temsirolimus [147] shows potent AD therapeutic effect in reducing p-Tau aggregates in P301S mice [148,149]. Polyphenol curcumin is a potent PI3K and mTOR inhibitor reported to alleviate AD pathology. Curcumin has restored spatial memory by inducing autophagic degradation of Aβ aggregates in APP/PS1 mice [150]. Curcumin has been extensively studied, and it is well understood to be an anti-AD drug. However, curcumin’s poor brain bioavailability prevents its clinical applications in AD [151,152,153,154,155,156,157]. Secoiridoid polyphenol oleuropein aglycone induces autophagy by inhibiting mTOR, and it activates AMPK by releasing Ca^2+^ from sarcoplasmic reticulum stores [158]. Oleuropein aglycone treatment in TgCRND8 mice has significantly reduced Aβ plaque density and restored synaptic plasticity by activating autophagy in a 12 month-old TgCRND8 mice model [159,160]. Methylene blue can induce autophagy similar to rapamycin and reduce the soluble and insoluble tau aggregates in the JNPL3 tau mice model [161]. However, in clinical trials, methylene blue appears to reduce the tau fibrils only while it increases granular tau oligomer levels [162].

#### 4.3.2. TFEB-Activating Aggrephagy Inducers

mTOR is considered to be one of the master regulators of protein synthesis. Aggrephagy inducers acting independently of mTOR inhibition will not alter protein synthesis. mTOR-independent TFEB activators and aggrephagy inducers have gained much attention recently. Under nutrient-rich conditions, mTOR inactivates autophagy-lysosomal biogenesis gene master regulator TFEB by phosphorylating it at ser-142 and ser-211 sites. TFEB phosphorylation, particularly at ser-211, allows 14-3-3 to bind to p-TFEB resulting in its subcellular localization [163,164]. However, under the starvation condition, TFEB is dephosphorylated and activated. Dephosphorylated TFEB can translocate into the nucleus and induce the transcription of the coordinated lysosomal expression and regulation (CLEAR) gene network. CLEAR gene encodes for the proteins necessary for the formation of autophagosomes, lysosomes, and factors involved in autolysosome formation [165,166,167,168]. Few compounds can promote TFEB mediated autophagy and lysosomal biogenesis to reduce the AD pathological aggregates without inhibiting mTOR activity. The natural disaccharide trehalose enhances TFEB nuclear translocation by inducing limited lysosomal membrane enlargement. It activates protein phosphatase-3 CB (PP3CB), calcineurin, and translocates TFEB into the nucleus, independent of inhibiting mTOR activity [169,170,171,172,173,174]. Trehalose treatment enhances the plaque clearance in APP/PS1 mice, and it reduces the tangle formation in P301S mice, again, independent of mTOR [170,171,173]. Ouabain is an mTOR and Na^+^/K^+^-ATPase inhibitor. It activates TFEB, reduces p-Tau aggregates, and restores cognitive deficits in P301L mice [175,176]. Cardiac glycoside-ingenol derivative Hep14 enhances the TFEB mediated ALP independent of mTOR inhibition. It promotes TFEB mediated ALP by activating PKC (both PKCα and PKCδ) as well as by inhibiting GSK3β. Hep14 induces TFEB nuclear translocation and reduces the plaques in APP/PS1 mice [177]. 5xFAD mice treatment with aspirin and cinnamic acid activates TFEB gene promotor PPARGC1α and increased TFEB expression level. Elevated TFEB promotes lysosomal biogenesis and reduces Aβ plaque burden [178,179]. 

## 5. Conclusions and Future Direction

AD is a type of proteinopathy. It is characterized by progressive accumulation of aberrant Abeta and p-Tau proteins aggregation in the brain, resulting in memory deficits and neurodegeneration. In AD, specific degradation of these aggregates, i.e., aggrephagy, is impaired due to altered substrate recognition, defects in axonal transport mechanism, and failure in lysosome acidification. The impaired aggrephagy process can be restored with aggrephagy enhancers. However, developing aggrephagy enhancers is a challenge because we still do not fully understand the aggrephagy mechanism in AD. For instance, the role of HDAC6 in Aβ and p-Tau aggresome formation, as well as their retrograde transport, and lysosomal degradation is still not clear. Molecular targets that can activate aggrephagy specifically need to be identified. The clearance of the conjugated aggrephagy receptor and aggregates complex is necessary to halt AD progression. In preclinical studies, small molecules targeted to promote ALP by inhibiting mTORC1 or by inducing TFEB nuclear translocation are efficacious in reducing AD aggregates as well as in alleviating memory dysfunction.

In a multifactorial disease like AD, translating clinical AD pathology into an experimental model is challenging. Currently, age-dependent AD transgenic mice models cannot accurately reproduce the clinical pathology of human AD [184,185]. Hence, in the development of an efficient anti-AD candidate to overcome the pre-clinical limitations, multiple transgenic mouse models with different disease phenotypes should be employed. Nevertheless, the clinical failure of aggrephagy inducer methylene blue highlights the importance of careful pre-clinical examination of future aggrephagy-enhancing compounds [162]. With proposed therapeutic agents, screening can eliminate off-target activation and validate their physiological relevance to alleviate the disease. Despite the drawbacks and limitations, developing aggrephagy inducers to alleviate AD pathology remains a promising strategy for improving AD therapy.

## Figures and Tables

**Table 1:** Aggrephagy enhancers for the treatment of AD.

Compound	Mode of Action	Animal Model	Dose/Route	Result	Reference
Curcumin	mTOR inhibition	APP/PS1	1000 ppm/P.O.	↓Aβ plaques, ↑Memory	[130,150]
Rapamycin	mTOR inhibition	PDAPP mice hAPP (J20) mice P301S mice	2.24 mg/kg/P.O. 2.24 mg/kg/P.O. 15 mg/kg/I.P.	↓Aβ plaques, ↑Memory ↓Cerebral amyloid angiopathy,↑Memory ↓Sarkosyl insoluble tau	[144,146,180]
Temsirolimus	mTOR inhibition	P301S mice Tg30 mice	20 mg/kg/I.P. 0.2 mg/kg/I.P.	↓Sarkosyl insoluble tau, ↑Memory ↓Sarkosyl insoluble tau, ↑Motor function	[148,181]
Oleuropein	AMPK activation, mTOR inhibition	TgCRND8 mice	50 mg/kg/P.O.	↓Aβ plaques, ↑Memory	[159]
Methylene blue	mTOR inhibition	JNPL3 mice	0.02 mg/kg/P.O.	↓Sarkosyl insoluble tau	[161]
Ouabain	mTOR inhibition, TFEB activation	P301L mice	1.5 µgm/kg/I.P.	↓Sarkosyl insoluble tau, ↑Memory	[176]
Trehalose	TFEB activation	P301S mice APP/PS1 mice	2% Trehalose in drinking water 5 µL injected into brain lateral ventricles	↓Sarkosyl insoluble tau, ↑Motor function ↓Aβ deposits, ↑Memory	[170,173,174]
Pseudoginsenoside-F11 (PF-11)	TFEB activation	SAMP8 mice	32 mg/kg/P.O.	↓Aβ plaques, ↑Memory	[182,183]
Hep-14	TFEB activation	APP/PS1 mice	5 mg/kg/I.P.	↓Aβ plaques	[177]
Aspirin	PPARGC1α mediated TFEB activation	5xFAD mice	2 mg/kg/P.O.	↓Aβ plaques	[179]
Cinnamic acid	PPARGC1α mediated TFEB activation	5xFAD mice	100 mg/kg/P.O.	↓Aβ plaques, ↑Memory, ↑Open field exploration	[178]

P.O: Oral route; I.P: Intraperitoneal route; PPM: parts per million.
